# Evaluation of Toluene Adsorption Performance of Mortar Adhesives Using Porous Carbon Material as Adsorbent

**DOI:** 10.3390/ma10080853

**Published:** 2017-07-26

**Authors:** Seunghwan Wi, Seong Jin Chang, Su-Gwang Jeong, Jongki Lee, Taeyeon Kim, Kyung-Won Park, Dong Ryeol Lee, Sumin Kim

**Affiliations:** 1 Building Environment & Materials Lab, School of Architecture, Soongsil University, Seoul 06978, Korea; dnltmdghks@ssu.ac.kr (S.W.); tjdwls329@ssu.ac.kr (S.J.C.); wjdtnrhkd@ssu.ac.kr (S.-G.J.); dlwhdrl123@ssu.ac.kr (J.L.); 2 Department of Architectural Engineering, Yonsei University, Seoul 03722, Korea; tkim@yonsei.ac.kr; 3 Department of Chemical Engineering, Soongsil University, Seoul 06978, Korea; kwpark@ssu.ac.kr; 4 Department of Physics, Soongsil University, Seoul 06978, Korea; drlee@ssu.ac.kr

**Keywords:** mortar adhesives, volatile organic compounds, small chamber, toluene, carbon nanomaterials

## Abstract

Porous carbon materials are advantageous in adsorbing pollutants due to their wide range of specific surface areas, pore diameter, and pore volume. Among the porous carbon materials in the current study, expanded graphite, xGnP, xGnP C-300, xGnP C-500, and xGnP C-750 were prepared as adsorbent materials. Brunauer–Emmett–Teller (BET) analysis was conducted to select the adsorbent material through the analysis of the specific surface area, pore size, and pore volume of the prepared porous carbon materials. Morphological analysis using SEM was also performed. The xGnP C-500 as adsorbent material was applied to a mortar adhesive that is widely used in the installation of interior building materials. The toluene adsorption performances of the specimens were evaluated using 20 L small chamber. Furthermore, the performance of the mortar adhesive, as indicated by the shear bond strength, length change rate, and water retention rate, was analyzed according to the required test method specified in the Korean standards. It was confirmed that for the mortar adhesives prepared using the xGnP C-500 as adsorbent material, the toluene adsorption performance was excellent and satisfied the required physical properties.

## 1. Introduction

In recent years, the need for improvement in the quality of residential environments has been increasing. In particular, the need for comfortable indoor air quality is increasing. Today, people spend 70 percent of their time indoors [1]. Therefore, their health can be directly linked to indoor air quality. Several types of indoor air pollutants have been detected in indoor environments. Formaldehyde and volatile organic compounds (VOCs) are typical pollutants. They are used as the main ingredients of paint solutions, silicon sealant, rubber, and various adhesives. In particular, VOCs are released from various building materials such as interior finishing materials, paints, and adhesives, and are then discharged into the indoor air. Therefore, it is important to use low-emission materials or to remove the pollutants through adsorption because they have a negative impact on the health of the human body [2,3]. Among the VOCs, toluene causes air pollution and damages human health, and can lead to severe neurological harm [4]. For this reason, research has been undertaken on methods to remove pollutants using adsorbents. The use of adsorbent is the basis of all adsorption techniques. Some porous materials such as graphene, activated carbon, and nanoporous carbon have been reported for VOC removal [5,6,7,8,9]. Also, porous materials that contain nanoscale porous material have been widely used to eliminate VOCs through their excellent adsorption capacities [10,11]. Furthermore, porous materials are advantageous in adsorption due to their high specific surface area and porosity [12,13]. The porosity and specific surface area of a material are critical factors in determining their adsorption performance for pollutants [14]. Surface area, pore volume, and pore size are the most important properties of porous materials, since these characteristics determine their adsorption performance. Brunauer–Emmett–Teller (BET) analysis, which is typically used to analyze these porous characteristics [15], is commonly based on nitrogen or argon adsorption isotherms at the boiling points of nitrogen and argon of 77 K and 87.15 K, respectively. However, CO_2_ may be used when measurement using nitrogen and argon isotherms is not possible [16]. Adhesive mortar is widely used to install interior finishing materials such as ceramic tiles and other decorative materials or polystyrene sheets [17,18]. The pollutants emitted from an adhesive mortar can directly affect indoor air quality. Since indoor air quality has become increasingly more important, the Korean government implemented the Construction Standard of Healthy-Friendly House in 2014. Appendix 6 of the Construction Standard of Healthy-Friendly House specifies the adsorption performance requirements for toluene and formaldehyde of building materials. The requirements are based on the adsorption rate and the total mass per area of sorption using the 20 L small chamber method, according to ISO 16000-23 and ISO 16000-24 [19,20]. For toluene adsorption performance, an adsorption rate of over 65% is required, and the total mass per area of sorption must be higher than 28,000 μg/m^2^. 

**Figure 1:**
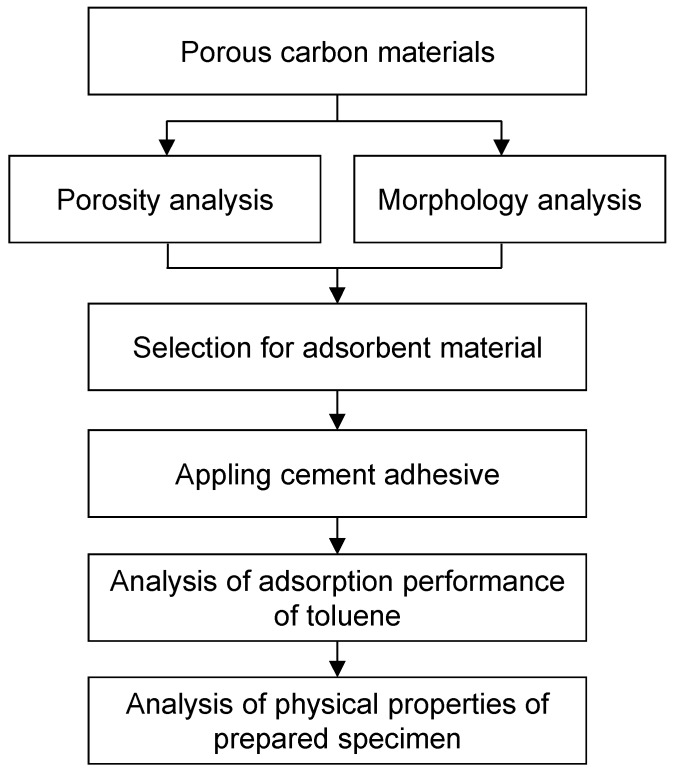
Flow diagram of the entire experiment.

In the preceding research, the adsorption performance of the raw material was mainly evaluated. However, in order to realize actual performance in residential space, it is necessary to study the case applied to building materials. In this study, the selection of the adsorbent materials was based on the porosity and specific surface area analysis of carbon materials using the Brunauer–Emmett–Teller (BET) equation. The adsorption material for the adhesive mortar was analyzed according to the toluene adsorption performance using a 20 L small chamber. The physical properties were tested according to the Korean Standard, KS L 1592 method [21]. The shear bond strength, length change rate, and water retention rate were analyzed for actual usability. Figure 1 shows a flow diagram of the entire experiment.

## 2. Experimental

### 2.1. Materials

Among the porous carbon materials, including expandable graphite (EG), exfoliated graphite nanoplatelets (xGnP), and C-grade xGnPs (xGnP C-300, xGnP C-500, and xGnP C-750), a total of five porous carbon materials were prepared for use as adsorbents. The EG and xGnP were arranged from sulfuric acid-intercalated expandable graphite (3772) obtained from Asbury Graphite Mills, Inc. (Asbury, NJ, USA) by applying the cost and time-effective exfoliation process initially proposed by Drzal’s group [22]. The C-grade xGnPs have different surface areas, ranging from about 300 m^2^/g to 750 m^2^/g. All of the C-grade xGnP materials have a small flake morphology, with particle sizes that are larger in the lower surface area materials and smaller in the higher surface area materials. The C-grade xGnPs were obtained from XG Science, Inc. (Grand Oak Drive, Lansing, MI, USA). Mortar adhesive was sourced to assess the performance of the toluene adsorption when applying the adhesive to a porous carbon material. The mortar adhesive (PA-7000) was obtained from SSANGKOM Corporation (Jung-gu, Seoul, Korea).

### 2.2. Test Method

#### 2.2.1. Porosity and Morphology Analysis for Adsorbent Material

All porous carbon materials were analyzed by BET (TriStar 3000, Micromeritics Instrument Corp., Communications Drive, Norcross, GA, USA) using an auto nitrogen adsorption instrument. In addition, to determine their porosity, the porous carbon materials were observed by scanning electron microscopy (JSM-6360A, JEOL, Dearborn Road, Peabody, MA, USA) at room temperature for morphological analysis. An SEM with an accelerating voltage of 12 kV and working distance of 12 mm was used to capture the SEM images. The samples were coated with a gold coating of a few nanometers thickness [23].

#### 2.2.2. Evaluation of Toluene Adsorption Performance Using a 20 L Small Chamber

After selecting the xGnP C-500 as adsorbent material through BET and SEM analysis, a common mortar adhesive widely used in interior building materials was added to the adsorbent material. Table 1 shows the mixing ratio of the xGnP C-500 as adsorbent material and mortar adhesive. The reference mortar adhesive without xGnP C-500 (MA/Ref.) and mortar adhesive with xGnP C-500 3 wt % (MA/x3) were dried for 48 h at 25 °C and 50% relative humidity. The toluene adsorption performance of the specimen was tested using a 20 L small chamber. The 20 L small chamber was developed in Japan to determine the emission levels of formaldehyde and VOCs from construction materials and paints, of which the performance [24] complies with the ASTM (D5116-97 and D6007-96) [25,26] and ECA (Nos. 2, 8, 13, and 16) [27,28,29,30]. The 20 L small chamber test was carried out according to ISO 16000-23 and ISO 16000-24 [19,20]. ISO 16000-23 and 24 specifies a general laboratory test method for evaluating the reduction in concentration of formaldehyde or volatile organic compounds (VOCs) by sorptive building materials. The sorption of pollutants can be brought about by adsorption, absorption, and chemisorption. The performance of the material, with respect to its ability to reduce the concentration of formaldehyde or VOCs in indoor air, is evaluated by measuring sorption flux and saturation mass per area. 

#### 2.2.3. Evaluation of Physical Properties of Adsorption Mortar Adhesive

The adhesive strength of mortar adhesive should be suitable for maintaining a constant strength when applying the adsorption material. Therefore, the physical properties of the mortar adhesive were analyzed both independently of the adsorbent material and when added to the adsorbent material. Table 1 shows the mixing ratio of the xGnP C-500 as adsorbent material and mortar adhesive for measuring the physical properties. Table 2 shows the composition of the ingredients of the mortar adhesive. The shear bond strength, length change rate, and water retention rate were measured. The test was performed according to the Korean Standard, KS L 1592 method [19].

## 3. Results and Discussion

### 3.1. Porosity and Morphology Analysis of Porous Carbon Material for Adsorbent Material

**Figure 2:**
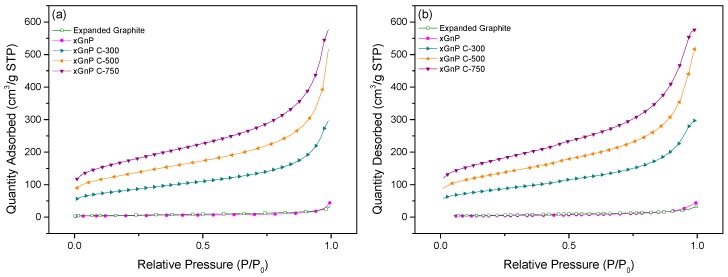
Adsorption–desorption isotherms of N_2_ at 77 K: (**a**) adsorption and (**b**) desorption.

**Figure 3:**
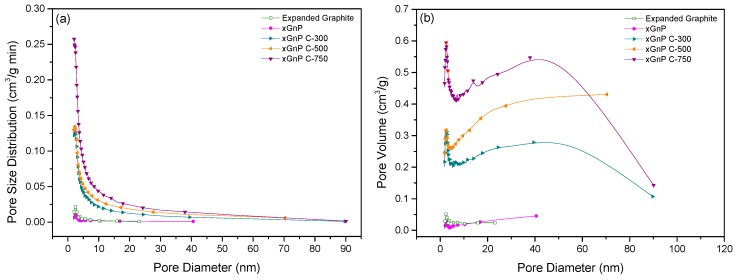
Pore diameter isotherms of porous carbon materials: (**a**) pore size distribution and (**b**) pore volume.

The specific surface area, pore diameter, and pore volume of the porous carbon materials were analyzed by BET analysis. The porous carbon materials were characterized by N_2_ adsorption at 77 K using a Micromeritics model TriStar 3000 analyzer and were outgassed for 24 h at 573 K to remove any moisture or adsorbed contaminants present on their surface. Figure 2a,b shows the selected adsorption and desorption isotherms of N_2_ at 77 K for porous carbon materials, respectively. The xGnP and EG were determined to have low adsorption amounts compared with the other porous materials. The xGnP C-300 grade, xGnP C-500 grade, and xGnP C-750 grade materials tended to increase gradually according to the grade. The xGnP C-750 showed the highest level of adsorption and indicated a constant amount of adsorption during the pressure change. Figure 3a,b shows the pore size distribution and pore volume of each porous carbon material. The porous carbon materials have a narrow pore size distribution. The pore sizes were mainly distributed below 10 nm, indicating that the porous carbon materials have a mesoporous pore diameter. The pore volume can be calculated from Equation (1). Figure 3b shows the calculated pore volume. xGnP C-750 has the largest pore volume and xGnP has the lowest.

(1) $$\mathrm{Pore}\;\mathrm{volume}=\underset{Pore\;diameter}{\sum }Pore\;size\;distribution$$

**Figure 4:**
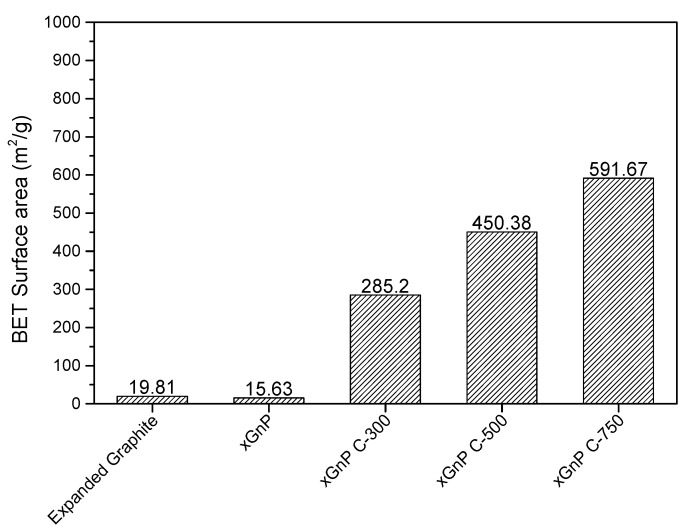
BET surface area of each porous carbon material.

**Figure 5:**
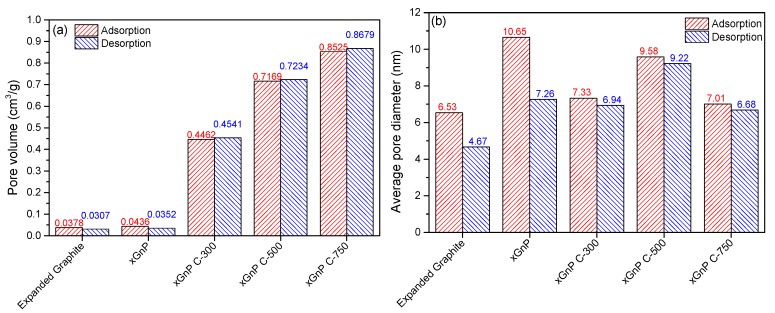
Adsorption and desorption properties of porous carbon materials of N_2_ at 77K: (**a**) pore volume and (**b**) average pore diameter.

Figure 4 shows the BET surface area of each porous carbon material. The xGnP C-750 had the widest specific surface area of 591.67 m^2^/g, while the xGnP had the narrowest specific surface area of 15.63 m^2^/g. The EG, xGnP C-300 grade, xGnP C-500 grade, and xGnP C-750 grade materials had specific surface areas of 19.81, 285.20, 450.38, and 591.67 m^2^/g, respectively. Therefore, the xGnP C-grade carbon materials had excellent specific surface areas. Figure 5a,b shows the pore volume and average pore diameter, respectively, in the adsorption and desorption of porous carbon materials. The pore volumes of the xGnP C-500 grade and xGnP C-750 grade carbon materials were 0.7169 and 0.8525 cm^3^/g in adsorption and 0.7234 and 0.8679 cm^3^/g in desorption, respectively. The average pore diameter of xGnP C-500 was 9.58 nm in adsorption and 9.22 nm in desorption. From these measurement results, the average pore size of xGnP C-500 grade was approximately 137.3% that of xGnP C-750. However, the pore volume of xGnP C-500 grade was approximately 83.7% that of xGnP C-750. From the BET analysis, the xGnP C-500 grade had excellent pore diameter, pore volume, and specific surface area. Therefore, xGnP C-500 was selected as the adsorbent material.

**Figure 6:**
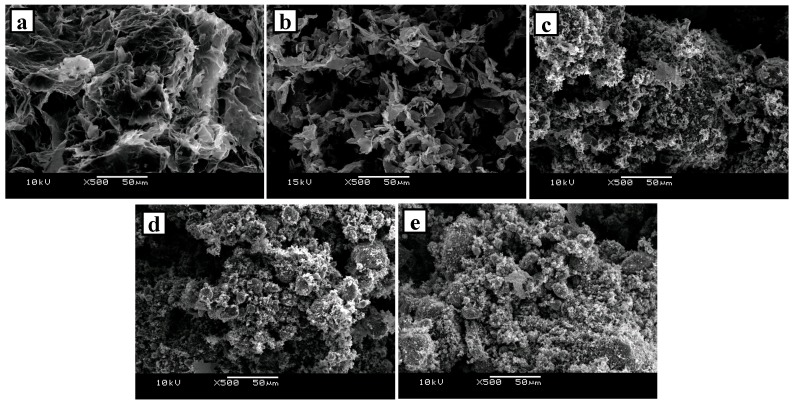
SEM image of porous carbon materials: (**a**) EG; (**b**) xGnP; (**c**) xGnP C-300; (**d**) xGnP C-500; and (**e**) xGnP C-750.

The morphology and microstructure of the carbon materials were analyzed by scanning electron microscopy (SEM, JEOL JSM-6360A). Figure 6 shows SEM images of all the porous carbon materials tested. xGnP C-300, xGnP C-500, and xGnP C-750 had a small microstructure, indicating the materials showed the same tendency as that for the specific surface area. The morphology analysis revealed that the porous carbon material having a large specific surface area has many mesoporous pores, as shown in the SEM image in Figure 6, and a good toluene adsorption performance is thus expected according to the microstructure of the porous carbon materials.

### 3.2. Evaluation of Toluene Adsorption Performance Using 20 L Small Chamber

**Figure 7:**
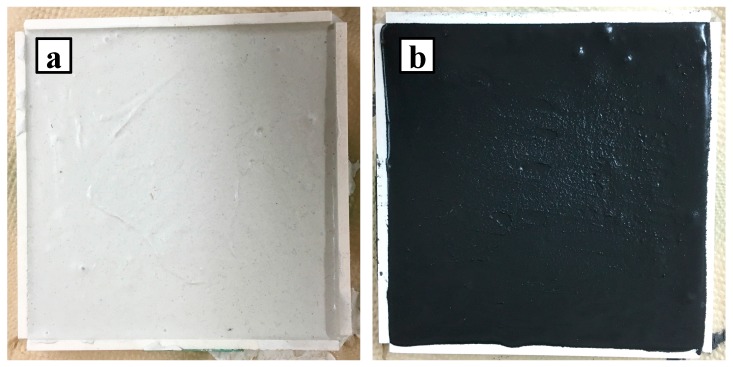
Manufactured mortar adhesive specimen: (**a**) MA/Ref. and (**b**) MA/x3.

**Figure 8:**
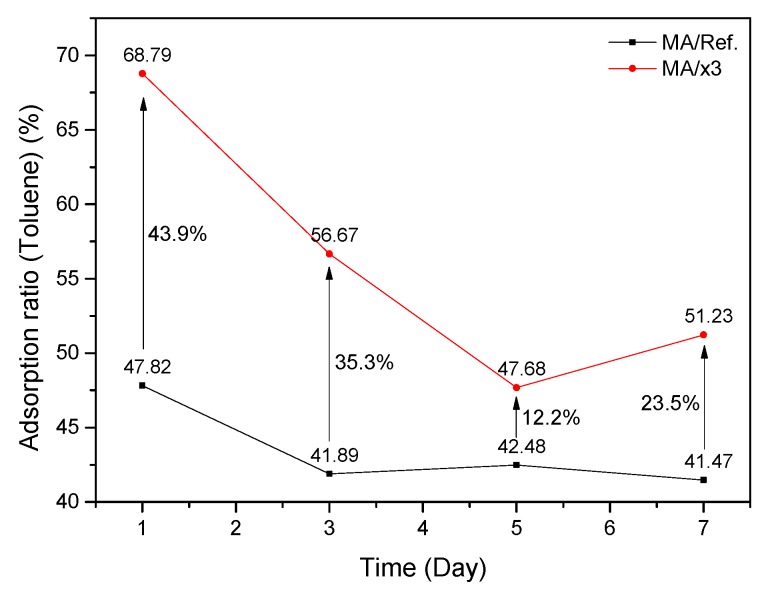
Adsorption ratio of toluene.

**Figure 9:**
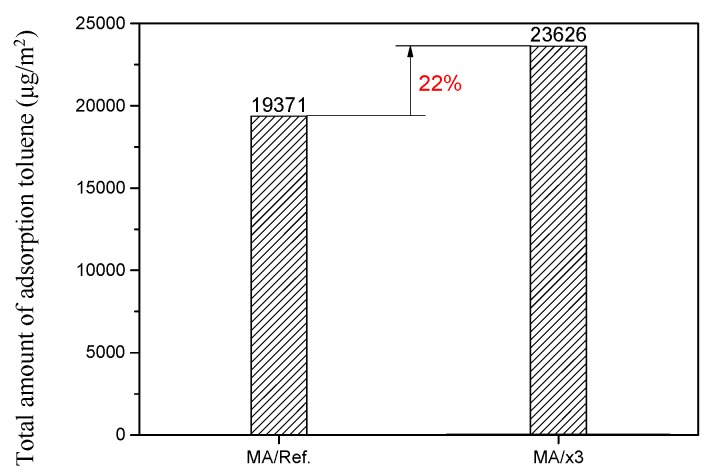
Total amount of adsorption toluene.

The toluene adsorption performance of the adsorbent material was tested using the 20 L small chamber method. Table 3 shows the test conditions. The mortar adhesive specimens were prepared to test the adsorption amount of toluene. Figure 7 shows the developed specimen. The test specimens prepared by varying the mixing ratio of xGnP C-500 grade were used to evaluate the adsorption performance of the adsorbent material. Figure 8 shows the adsorption rate of toluene. The adsorption rate is calculated using Equation (2) as follows.

(2) $$R_{a}=\left[(C_{in,t}-C_{out,t}\right)/C_{in,t}]\times 100$$

where $R_{a}$ is the adsorption rate (%), $C_{in,t}$ is the supply air concentration with elapsed time (μg/m^3^), and $C_{out,t}$ is the exhaust air concentration with elapsed time (μg/m^3^). The difference between the supply air concentration and exhaust air concentration can determine the amount of toluene contaminant the specimen has adsorbed. The emission rate of MA/Ref. and MA/x3 were 5.936 and 26.137 μg/m^2^·h, respectively. The adsorption ratio of MA/Ref. after 1, 3, 5, and 7 days was 47.82, 41.89, 42.48, and 41.47%, respectively. However, the adsorption ratio of MA/x3 after 1, 3, 5, and 7 days was 68.79%, 56.67%, 47.68%, and 51.23%, respectively. As emission rate is high, the result of MA/x3 may be different from the trend of general adsorption ratio. The toluene adsorption rate increased by 28.7% on average after seven days, implying that the xGnP C-500 grade material improves the adsorption performance of the mortar adhesive.

(3) $$F=\left(C_{in,t}-C_{out,t}\right)\times Q_{c}/A$$

(4) $$S_{c}=\underset{i}{\sum }\left(F_{i}\times ∆T_{e,i}\right)$$

where F is the adsorption flux (μg/m^2^·h), $C_{in,t}$ is the supply air concentration with elapsed time (μg/m^3^), $C_{out,t}$ is the exhaust air concentration with elapsed time (μg/m^3^), $Q_{c}$ is the ventilation rate of the chamber (m^3^/h), A is the surface area of the specimen (m^2^), $S_{c}$ is the total amount of adsorption (μg/m^2^), and $T_{e}$ is the elapsed time. Figure 9 shows the total amount of toluene adsorption, where the total amount of toluene adsorption mixed with 3 wt % of the xGnP C-500 grade specimen, MA/x3 was 23,626 μg/m^2^ and the total amount of toluene adsorption without the xGnP C-500 grade specimen, MA/Ref. was 19,371 μg/m^2^. The adsorption performance of the specimen mixed with xGnP C-500 grade as the adsorbent material was improved by about 22%, which means that the toluene adsorption performance of the mortar adhesive had significantly improved.

### 3.3. Evaluation of Physical Properties of Adsorption for Mortar Adhesive

An experiment was conducted in order to verify the fundamental physical properties of the mortar adhesive for the shear bond strength, the length change rate, and the water retention rate when mixed with the xGnP C-500 as adsorbent material. The measurements were carried out according to the Korean Standard, KS L 1592:2011 [21]. Table 4 shows the test results of the mortar adhesive. The shear strengths were measured three times: 7 days air dry curing, 28 days air dry curing, and 28 days air dry curing after freezing and thawing. The measured shear bond strengths without the adsorbent material, MA/Ref. were 1.86, 2.25, and 1.75 N/mm^2^ after 7 days air dry curing, 28 days air dry curing, and 28 days air dry curing, respectively, after freezing and thawing. The length change rate and water retention rate of MA/Ref. were 0.15% and 89%, respectively. However, the measured shear bond strengths of the mortar adhesive mixed with the adsorbent material, MA/x3 were 1.10, 1.23, and 0.72 N/mm^2^ after 7 days air dry curing, 28 days air dry curing, and 28 days air dry curing after freezing and thawing, respectively. The length change rate and water retention rate of MA/x3 were 0.11% and 93%, respectively. As a result, the shear bond strength of mortar adhesive decreased when the mortar adhesive was mixed with the adsorbent material. The required physical performance of KS L 1592 was satisfied, even though the adhesive strength had decreased.

## 4. Conclusions

Porous carbon materials are widely used as adsorbent materials for adsorbing pollutants due to their excellent porosity and their wide range of specific surface areas, pore diameter, and pore volume. Therefore, the pore diameter, pore volume, and specific surface area were analyzed using BET to select the adsorbent material. Among the porous carbon materials tested, xGnP C-500 had better pore diameter, pore volume, and specific surface area as adsorbent material than other porous carbon materials. The porous properties of xGnP C-500 grade was 450.38 m^2^/g of BET surface area, 0.7169 cm^3^/g of pore volume in adsorption and 9.58 nm of average pore diameter, respectively. The selected xGnP C-500 as an adsorbent material was mixed with a commonly used mortar adhesive; mortar adhesive is widely used in the interior finishing of building materials, and is known to contain various pollutants. The toluene adsorption performance of the mortar adhesive when mixed with the adsorbent material, xGnP C-500, was evaluated using a 20 L small chamber test according to ISO 16000-23 and 24 [19,20]. It was confirmed that the toluene adsorption performance of the mortar adhesive mixed with xGnP C-500 improved by 28.7% of toluene adsorption rate after seven days on average and 22% of total amount of adsorption toluene, respectively. Therefore, it is possible to reduce the concentration of toluene released from finishing of building materials in the construction stage using xGnP C-500 grade as adsorbent. The physical properties of the prepared specimen using adsorbent material were also analyzed. The measurements were carried out according to the Korean Standard, KS L 1592:2011 [21]. The results of the required physical performance specified in KS L 1592:2011 [21] was satisfied of all tested items, even though the adhesive strength had decreased. Consequently, it was confirmed that the mortar adhesives prepared using the xGnP C-500 as adsorbent material improved the adsorption performance of toluene and satisfied the required physical properties. In further study, adsorption performance of various xGnP C grades for application of building materials will be studied.

## Figures and Tables

**Table 1:** Mixing ratios of specimens.

Sample	Mortar Adhesive (g)	Water (g)	xGnP C-500 (g)
Mortar adhesive (MA/Ref.)	1	0.45	0
Mortar adhesive/xGnP C-500 3 wt % (MA/x3)	1	0.45	0.03

**Table 2:** Composition of ingredients of mortar adhesive.

Ingredients	CAS No.	% by Weight
Dolomite	16389-88-1	45–55
Portland cement	65997-15-1	35–45
Redispersible polymer powder	-	4–8
Methyl cellulose	9004-67-5	0.1–0.5

**Table 3:** Test conditions in the 20 L small chamber.

Test Condition	20 L Small Chamber
Sample area (m^2^)	0.0392
Volume (L)	20
Loading factor (area of sample/volume, m^2^/m^3^)	1.96
Air change rate (h^−1^)	0.5
Flow rate (mL/min)	1.0
Injection volume (μL)	25
Equilibration time (hour)	24, 72, 120, 168
Temperature (°C), Relative humidity (%)	25 ± 1.0, 50 ± 5
Background concentration (μg/m^3^)	HCHO: <2, TVOC: <20
Analysis method	VOC: GC/MS

**Table 4:** Test results of mortar adhesive.

Test Items	Unit	Reference Mortar Adhesive (MA/Ref.)	Mortar Adhesive with xGnP C-500 3 wt % (MA/x3)	Korean Standards (KS L 1592:2011)
Shear bond strength (7 days air dry curing)	N/mm^2^	1.86	1.10	More than 1.03
Shear bond strength (28 days air dry curing)	N/mm^2^	2.25	1.23	More than 1.03
Shear bond strength (28 days air dry curing after freezing and thawing)	N/mm^2^	1.75	0.72	More than 0.69
The length change rate	%	0.15	0.11	Below 0.2
The water retention rate	%	89	93	From 80 to 95

## References

[1] Jones A.P. (1999). Indoor air quality and health. Atmos. Environ..

[2] Kim S., Kim H., Moon S. (2006). Evaluation of VOC emissions from building finishing materials using a small chamber and VOC analyser. Indoor Built Environ..

[3] Kim K., Kim S., Kim H., Park J.C. (2010). Formaldehyde and TVOC emission behaviors according to finishing treatment with surface materials using 20 L chamber and FLEC. J. Hazard. Mater..

[4] Page N.P., Mehlman M. (1989). Health effects of gasoline refueling vapors and measured exposures at service stations. Toxicol. Ind. Health.

[5] Shiue A., Kang Y., Hu S., Jou G., Lin C., Hu M., Lin S. (2010). Vapor adsorption characteristics of toluene in an activated carbon adsorbent-loaded nonwoven fabric media for chemical filters applied to cleanrooms. Build. Environ..

[6] Anbia M., Khataei N.K. (2012). Ordered nanoporous carbon as an effective adsorbent in solid-phase microextraction of toluene and chlorinated toluenes in water samples. J. Saudi Chem. Soc..

[7] Zhang W., Zhang L.Y., Zhao X.J., Zhou Z. (2016). Citrus pectin derived porous carbons as a superior adsorbent toward removal of methylene blue. J. Solid State Chem..

[8] Razali M., Kim J.F., Attfield M., Budd P.M., Drioli E., Lee Y.M., Szekely G. (2015). Sustainable wastewater treatment and recycling in membrane manufacturing. Green Chem..

[9] Fu C., Wang Z., Liu J., Jiang H., Li G., Zhi C. (2016). Large scale fabrication of graphene for oil and organic solvent absorption. Prog. Nat. Sci. Mater. Int..

[10] Seo J., Kato S., Ataka Y., Yang J. (2010). Influence of environmental factors on performance of sorptive building materials. Indoor Built Environ..

[11] Liu C., Tang Z., Chen Y., Su S., Jiang W. (2010). Characterization of mesoporous activated carbons prepared by pyrolysis of sewage sludge with pyrolusite. Bioresour. Technol..

[12] Huang Q., Vinh-Thang H., Malekian A., Eić M., Trong-On D., Kaliaguine S. (2006). Adsorption of n-heptane, toluene and o-xylene on mesoporous UL-ZSM5 materials. Microporous Mesoporous Mater..

[13] Ryu Y., Lee H., Yoo H., Lee C. (2002). Adsorption equilibria of toluene and gasoline vapors on activated carbon. J. Chem. Eng. Data.

[14] Lee W., Park J., Sok J., Reucroft P. (2005). Effects of pore structure and surface state on the adsorption properties of nano-porous carbon materials in low and high relative pressures. Appl. Surf. Sci..

[15] Kim K.C., Yoon T., Bae Y. (2016). Applicability of using CO_2_ adsorption isotherms to determine BET surface areas of microporous materials. Microporous Mesoporous Mater..

[16] Chui S.S., Lo S.M., Charmant J.P., Orpen A.G., Williams I.D. (1999). A chemically functionalizable nanoporous material. Science.

[17] Petit J., Comelli B., Perrin R., Wirquin E. (2016). Effect of formulation parameters on adhesive properties of ANSI 118–15 and 118–11 compliant tile adhesive mortars. Int. J. Adhes. Adhes..

[18] Petit J., Wirquin E. (2013). Evaluation of various cellulose ethers performance in ceramic tile adhesive mortars. Int. J. Adhes. Adhes..

[19] (2009). Indoor Air—Part 23: Performance Test for Evaluating the Reduction of Formaldehyde Concentrations by Sorptive Building Materials.

[20] (2009). Indoor Air—Part 24: Performance Test for Evaluating the Reduction of Volatile Organic Compound (Except Formaldehyde) Concentrations by Sorptive Building Materials.

[21] (2011). Cement for Ceramic Tiles.

[22] Kim S., Drzal L.T. (2009). High latent heat storage and high thermal conductive phase change materials using exfoliated graphite nanoplatelets. Sol. Energy Mater. Sol. Cells.

[23] Lee J., Kim J., Kim S., Kim J.T. (2013). Thermal extractor analysis of VOCs emitted from building materials and evaluation of the reduction performance of exfoliated graphite nanoplatelets. Indoor Built Environ..

[24] Lee J., Kim S. (2012). The determination of the adsorption performance of graphite for VOCs and formaldehyde. Energy Build..

[25] (1997). Standard Guide for Small-Scale Environmental Chamber Determinations of Organic Emissions from Indoor Materials/Products.

[26] (1996). Standard Test Method for Determining Formaldehyde Factors in Air from Wood Products Using a Small Scale Chamber.

[27] (1989). Guideline for the Determination of Steady State Concentrations in Test Chambers.

[28] (1991). Guideline for the Characterization of Volatile Organic Compounds Emitted from Indoor Materials and Products Using Small Test Chambers.

[29] (1993). Determination of VOCs Emitted from Indoor Materials and Products—Inter Laboratory Comparison of Small Chamber Measurements.

[30] (1995). Determination of VOCs Emitted from Indoor Materials and Products—Second Inter Laboratory Comparison of Small Chamber Measurements.

